# Enhancement of Self-Collimation Effect in Photonic Crystal Membranes Using Hyperbolic Metamaterials

**DOI:** 10.3390/nano12030555

**Published:** 2022-02-06

**Authors:** Yaoxian Zheng, Qiong Wang, Mi Lin, Zhengbiao Ouyang

**Affiliations:** THz Technical Research Center of Shenzhen University, Shenzhen Key Laboratory of Micro-Nano Photonic Information Technology, Key Laboratory of Optoelectronic Devices and Systems of Ministry of Education and Guangdong Province, College of Physics and Optoelectronic Engineering, Shenzhen University, Shenzhen 518060, China; jewel5282@163.com (Y.Z.); qwang@szu.edu.cn (Q.W.); linfengas111@szu.edu.cn (M.L.)

**Keywords:** hyperbolic metamaterials, self-collimation effect, photonic crystals, beam behavior

## Abstract

Hyperbolic metamaterials (HMMs) exhibit high tunability in photonic devices. This study numerically investigates light propagation in photonic crystal (PhC) membranes containing HMMs. The proposed HMM PhC membranes contain square HMM rods, which comprise dielectric (Si) and metallic (Ag) layers. Owing to their property of subwavelength field localization, HMMs can be applied to PhCs to improve tunability and thus enhance the self-collimation (SC) effect of PhCs. The SC points were obtained in the second HMM PhC band, wherein the nearby dispersion curves change significantly. In addition, the effect of the HMM filling factor (i.e., the ratio of the metal-layer to unit-cell thicknesses) on the SC point frequency is studied. Finally, we demonstrate the efficient control of beam behaviors using HMM PhC membranes while considering the nonlinearity of Ag. The findings of this study confirm that high-performance HMM PhC membranes can be employed in nonlinear all-optical switches, filters, tunable lenses, and other integrated optical devices.

## 1. Introduction

Photonic crystals (PhCs) are periodic spatial variations in dielectric permittivity on the wavelength scale [1,2,3]. The periodicity allows PhCs to acquire unique dispersion properties, which originate from Bragg scattering by the nanorods inside PhCs. For such periodic systems, we can investigate their Bloch modes, which are important to achieve many excellent properties of PhCs. Over the past decade, PhCs have attracted increasing research attentions, owing to their fascinating abilities. PhCs have become essential parts for novel nanophotonic devices [4,5,6,7]. The phenomena of PhCs include: total reflection from their bandgaps, negative refraction [8], and super-prism effect [9]. One of the dispersion properties of PhCs is a self-collimation (SC) effect. In a PhC that support the SC effect, the energy flow of waves that propagate inside the PhC can remain in one direction. Without any additional structure, a PhC can support waves propagating with field confinement, acting as a waveguide. Most SC effects only support a limited range of the angle of the incident beam. However, one kind of SC effect can support the SC propagation of waves from any angle, which is called an all-angle SC effect. Having an all-angle SC property means that they will have the strongest field confinement, and the mode volume can achieve the smallest level. When SC effects have low group velocities, they can be utilized as high quality-factor (Q-factor) waveguides and cavities. Usually, low group velocity brings high tunability, as well as effective modulation of the beam behavior of PhCs [10,11,12].

Metamaterials are another artificial material with subwavelength patterns [13,14,15,16]. Usually, they exhibit average effects of the electromagnetic response of the medium. One branch of metamaterials is hyperbolic metamaterials (HMMs), which comprise layered metal-dielectric structures [17,18,19,20]. HMMs can show high anisotropic properties, owing to the presence of the opposite components of effective electric tensors. Moreover, both PhCs and HMMs exhibit patterns, and both are excellent candidates for high-performance optical devices. Many researchers have focused on bringing them together. PhCs formed by HMMs are expected to exhibit unique and excellent properties. Those properties include: omnidirectional bandgaps [21], Goos–Hanchen shift [22], and photonic hypercrystallinity [23,24,25].

Most previous studies have invariably focused on one-dimensional (1D) PhCs with HMMs [26,27,28]. However, in wave propagation, two-dimensional (2D) PhC membranes are reckoned to afford wide-scale prospects concerning the realization of 2D bandgaps that can be used in the development of high-Q-factor 2D waveguides and/or resonant cavities [29,30]; and 2D PhCs demonstrate outstanding dispersion properties. They can be used to control wave propagation and beam behavior in the second dimension, and this makes them superior to 1D PhCs [31,32,33]. Moreover, HMMs could be used to improve the tunability of conventional PhC devices as well as achieve high efficiency and realize novel functionalities [34,35]. Therefore, HMMs can be used to enhance all-angle SC effects and facilitate the design of efficient methods for controlling light propagation in PhCs.

Extant studies have seldom investigated all-angle SC effects in HMM PhC membranes. Optical cavities have strong field confinements, and thus their tunabilities can reach very high levels. But the cavities will lose their flexibilities. Especially when manufacturing defects exist, cavity systems would fail. The SC effects of 2D PhCs, however, provide another method for achieving field confinements, while retaining flexibilities, since SC effects are overall effects, and can be immune to local defects. However, it is difficult to achieve high tunability for such overall effects. In this context, we propose the construction of PhC membranes using HMMs to improve tunability, increase the efficiency of modulating the dispersion property, and control wave propagation. In this study, we numerically assessed the dispersion properties and band structures of HMM-based PhC membranes to investigate their unique properties in controlling light propagation and realizing all-angle SC operating modes. To this end, we first obtained the band structures of the proposed HMM PhC membranes, considering specific structural parameters. Operating at the SC point, the proposed HMM PhC membranes can support all-angle low-group-velocity SC effects, which facilitate their use as high-Q-factor waveguides and cavities. Second, we investigated the beam behaviors of divergence, collimation, and convergence during light propagation in the HMM PhC membranes. The change in the HMM filling factor—i.e., the ratio of the metal-layer to unit-cell thicknesses—at the SC frequency was investigated. Third, we observed that the effective permittivity of HMMs depends significantly on the operating frequency. These findings reveal that compared to PhC membranes with conventional dielectric rods, HMM PhCs are more efficient at modulating the dispersion property and controlling wave propagation in the system near the SC point. Finally, we investigated the modulation of beam behavior by the proposed HMM PhC membranes by considering the nonlinearity of Ag. The findings of this investigation confirm the high tunability of HMM PhC membranes in SC modes, thereby warranting their use in switching applications. Therefore, the HMM PhC membranes with all-angle SC modes are highly promising for use in several optical devices, including all-optical switches, filters, and tunable lenses.

## 2. Materials and Methods

**Figure 1:**
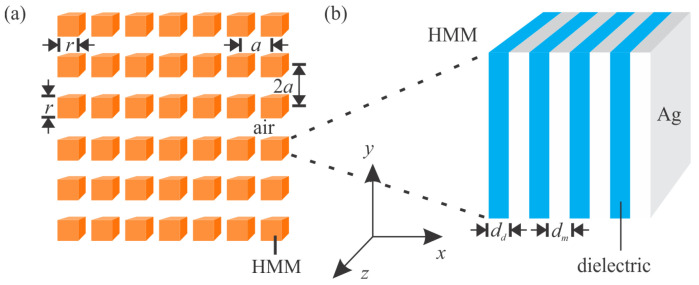
(**a**) Schematic of PhC membranes containing HMM square rods. (**b**) Schematic of HMMs composed of dielectric layers and Ag layers.

The proposed PhC membrane consists of HMM square rods inside the background medium of air (*n*_0_ = 1). As shown in Figure 1a, the *x* and *y* components of the rectangular lattice of the PhC are *a* = 0.2 μm and 2*a* = 0.4 μm, respectively, and the side length of the HMM rods is *r* = 0.5 × *a*. Because typical HMM structures consist of metal and dielectric layers, it would be considered appropriate to use the experimental refractive index data. However, for convenience and universality, we use the Drude model to describe the permittivity of Ag and use a constant to present the dielectric medium. The Drude model provides a simplification that facilitates the calculations. In the HMM PhC system, the SC effect can be very sensitive to permittivity, which makes it difficult to complete experiments. Using the Drude model will aggravate the deviation between theory and practice. Fortunately, the influence here is relatively little. For metal layers, the permittivity of Ag can be calculated from the Drude model as follows [36]:

(1) $$\varepsilon_{m}=\varepsilon_{\infty }-\frac{\omega_{p}^{2}}{\omega (\omega +i\gamma )}$$

where *ε*_m_ is the high-frequency permittivity, *γ* is the damping term, and *ω_p_* is the plasma angular frequency. From experimental data of Ag [37], $\varepsilon_{\infty }$ = 1.4447, *γ* = 9.1269 × 10^13^ rad/s, and *ω_p_* = 1.328 × 10^16^ rad/s. These parameters are obtained by curve fitting of the experimental data. For dielectric layers, the refractive index and extinction coefficient of the dielectric medium were set as *n*_Si_ = 4.1020 and *k*_Si_ = 0.043853, respectively, corresponding to the refractive index and extinction coefficient of Si at a frequency of 550 THz. The arrangement of the multilayer structure of the HMM is shown in Figure 1b. The thicknesses of the metal and dielectric layers are *d*_m_ and *d*_d_, respectively. The metal-to-dielectric layer thickness ratio (*d*_m_/*d*_d_) can be changed to modify the properties of the HMMs. There exist 20 layers within a single HMM rod, and the thickness of the two layers as a period is *d*_m_ + *d*_d_ = 10 nm; thus, the HMM rods are square shaped with a side length of *r* = 0.1 μm.

Because the HMM layer thickness is much smaller than the wavelength, an effective medium approximation can be used to investigate the PhCs containing multilayer HMM rods. Through further investigation, we found that 20 layers is sufficient for metamaterial approximation. Using the effective medium model, the dielectric components in the directions parallel and perpendicular to the incident wave vector can be written as follows [38,39]

(2) $$\varepsilon_{xx}=\frac{\varepsilon_{m}\varepsilon_{d}}{(1-f)\varepsilon_{m}+f\varepsilon_{d}}$$

(3) $$\varepsilon_{yy}=\varepsilon_{zz}=f\varepsilon_{m}+(1-f)\varepsilon_{d}$$

where *ε_xx_* and *ε_yy_* are the transverse and longitudinal components of the effective permittivity of the HMM, respectively. In Equations (2) and (3), *ε*_m_ and *ε*_d_ are the permittivities of the metal (Ag) and dielectric (Si), respectively. The permittivity of Ag is given by the Drude model in Equation (1). The coefficient *f* in Equations (2) and (3) is the ratio of the metal-layer thickness to the total thickness of a unit-cell of HMM, defined as the filling factor:

(4) $$f=\frac{d_{m}}{d_{d}+d_{m}}$$

For *H*_z_ polarization, the effective permittivity of HMMs is anisotropic, and the signs of its transverse and longitudinal components could be positive, negative, or zero. Thus, HMMs can be classified as effective dielectric (*ε_xx_* > 0, *ε_yy_* > 0), effective metal (*ε_xx_* < 0, *ε_yy_* < 0), Type I HMM (*ε_xx_* < 0, *ε_yy_* > 0), and Type II HMM (*ε_xx_* > 0, *ε_yy_* < 0) [40]. Different types of HMMs can support different electromagnetic modes in a system. In a PhC system, when HMM rods are applied instead of conventional dielectric rods in unit cells, the dispersion property of the entire PhC is different. Unique modes in PhCs can be generated when the operating type of HMM is Type I or Type II because such materials differ from both dielectrics and metals. Moreover, light propagation in HMM PhC systems is highly complicated. In bulk HMM systems, the effective dielectric and Type II HMM can support the propagation of incident light; in the effective metal and Type I HMM, propagating waves result in total reflections because no real wave vector satisfies the dispersion relation. Thus, HMM PhCs are periodic HMM systems because of the complicated diffraction of light by HMM rods, and Bloch modes would be unique.

**Figure 2:**
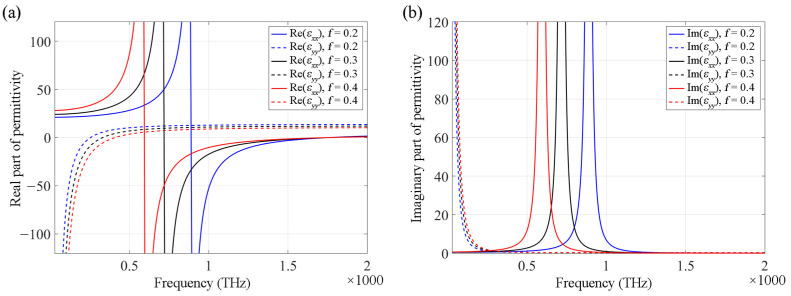
Transverse (solid line) and longitudinal (dashed line) components of effective permittivity of HMMs with different filling factors of *f* = 0.2 (blue), *f* = 0.3 (black) and *f* = 0.4 (red), respectively, at different frequencies. (**a**,**b**) show the real and imaginary parts of the effective permittivity, respectively.

According to Equations (2) and (3), the transverse and longitudinal components of the permittivity at various frequencies are shown in Figure 2. At different frequencies, the HMM has different optical properties. For instance, when the operating frequency is less than 400 THz, the HMM with *f* = 0.4 functions as a Type II HMM; when the operating frequency is between 420 THz and 590 THz, the HMM with *f* = 0.4 functions as an effective dielectric; when the operating frequency is between 600 THz and 1750 THz, the HMM with *f* = 0.4 functions as a Type I HMM; when the operating frequency exceeds 1750 THz, the HMM functions as an effective dielectric again. As shown in the figure, by changing the HMM filling factor, the signs of the transverse and longitudinal components of the permittivity of HMMs can be changed, thereby modifying their optical properties. An HMM system can be adjusted to realize a specific type of performance. Moreover, the transition of the types of HMMs can provide high-performance optical switches. By calculating the band structures of the HMM PhCs, we can observe that the propagation modes of the PhCs are determined by the effective permittivity of the HMMs.

By replacing the dielectric or other bulk materials in a conventional PhC membrane with HMMs, the former can be made to support new operating modes, and therefore, unique dispersion properties can be realized. We numerically calculated the band structure of an HMM PhC membrane and investigated the light propagating in the PhC. Band structures and wave propagation properties were calculated using the commercial software COMSOL, where only **H**_z_ polarization was considered. The effective permittivity of the HMM is an anisotropic tensor given by

(5) $$\varepsilon_{\mathrm{HMM}}=\left(\begin{array}{ccc} \varepsilon_{xx} & 0 & 0\\ 0 & \varepsilon_{yy} & 0\\ 0 & 0 & \varepsilon_{zz} \end{array}\right)$$

where *ε_xx_*, *ε_yy_*, and *ε_zz_* are given by Equations (2) and (3) described above. For *E*_z_ polarization, waves have the components of *H*_x_, *H*_y_ and *E*_z_. We can see that since we already have *E*_x_ = 0 and *E*_y_ = 0, the *x* and *y* components of the electric displacement vector **D** also are 0; thus, for **E**_z_ polarization waves only the *z* component of the permittivity tensor (Equation (5)) needs to be considered. For *H*_z_ polarization where *E*_z_ = 0, waves have the components of *E*_x_, *E*_y_ and *H*_z_. In this case, the *z* component of the permittivity tensor is useless since the *z* component of the electric displacement vector is 0. Moreover, the different *E*_x_ and *E*_y_ make the material with such an anisotropic tensor hyperbolic in *H*_z_ polarization. Thus, from Equation (5), for *E*_z_ polarization waves, we only need to consider *ε_zz_*, and the multilayers of metal and dielectric are no longer hyperbolic. Therefore, HMMs can operate as hyperbolic materials with *H*_z_ polarization waves exclusively. Accordingly, *H*_z_ polarization is exclusively investigated in this study.

## 3. Results and Discussion

### 3.1. Dispersion Properties and SC Modes of HMM PhC Membranes

**Figure 3:**
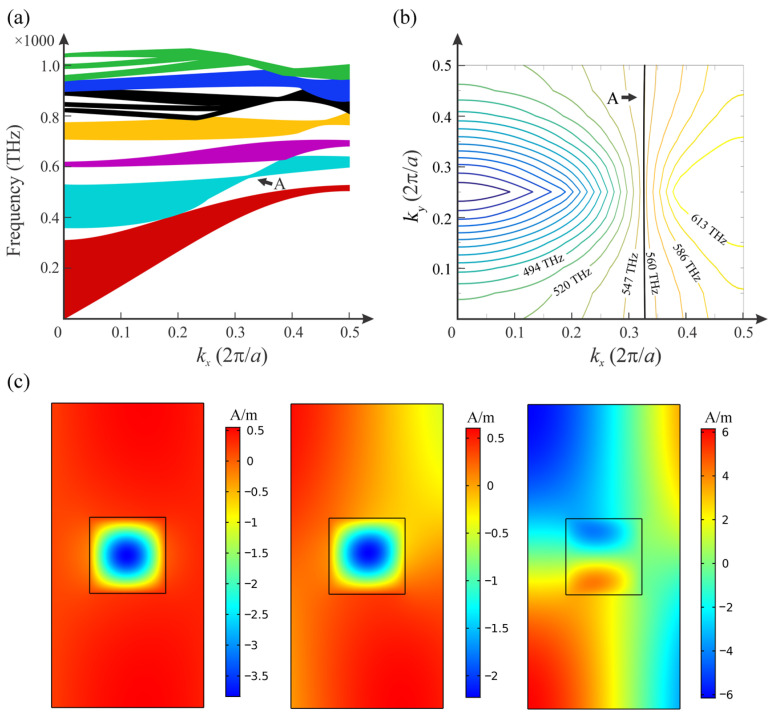
(**a**) Band structure of the HMM PhC membranes with filling factor of *f* = 0.2. Point A indicates an all-angle SC mode, (**b**) equifrequency contours of the second HMM PhC band, (**c**) magnetic field (|**H**|) profiles of point A with *k_y_* = 0, *k_y_* = 0.15 (2π/*a*) and *k_y_* = 0.25 (2π/*a*) in (**a**).

The band structure of the HMM PhC membrane was calculated by setting the filling factor to *f* = 0.2, as shown in Figure 3a. Note that Figure 3a is the projection of the three-dimensional band structure, which contains *k_x_*, *k_y_* and frequency, onto the *k_x_*-frequency plane. We have applied the filling factor of *f* = 0.2 to optimize the result. The SC point exists when the filling factor is between 0.1 and 0.45, which will be discussed later. In the frequency range where the HMM performs as an effective dielectric, the band structure of the HMM PhC membrane is similar to that of a conventional PhC membrane containing dielectric rods. However, at other frequencies, when the HMM performs as a Type I HMM or Type II HMM, the electromagnetic modes are significantly different and unique bands emerge. Here, we only focus on the second band, with point A, *f*_SC_ = 554.57 THz. Because the same frequency can have different *y* components of vectors (*k_y_*), the point has an all-angle SC effect for waves propagating along the *x*-axis. Figure 3b shows the equifrequency contours of the second band. The equifrequency contour of 554.57 THz is a flat contour, corresponding to the SC point of the band in Figure 3a. The flat equifrequency contour shows that the Bloch wave vectors are all in the same direction, explaining the occurrence of the all-angle SC effect. Furthermore, the sparse equifrequency contours in Figure 3b indicate a low-group-velocity; thus, the HMM PhC can be effective in increasing localized electromagnetic fields. The magnetic field profiles |**H**| of the SC point with different *k_y_* are shown in Figure 3c, which also depicts the field localization of the SC modes. Moreover, the shapes of the contours change significantly around the SC point; thus, light propagating in the PhC would be frequency-sensitive. When considering nonlinearity, the frequency-sensitive aspect results in high tunability of the all-optical functionalities.

According to Figure 3a, when the filling factor *f* = 0.2, the second band of the HMM PhC membrane is located in the frequency range of 350–630 THz, and the corresponding permittivity of HMM indicates that it is now functioning as an effective dielectric. The SC point arises at the frequency of 554.57 THz, and the transverse and longitudinal components of effective permittivity of the HMM at 554.57 THz are 30.99 + 0.42*i* and 10.85 + 0.08*i*, respectively. However, the generation of the SC point does not require the HMM to perform as an effective dielectric, which will be discussed later.

**Figure 4:**
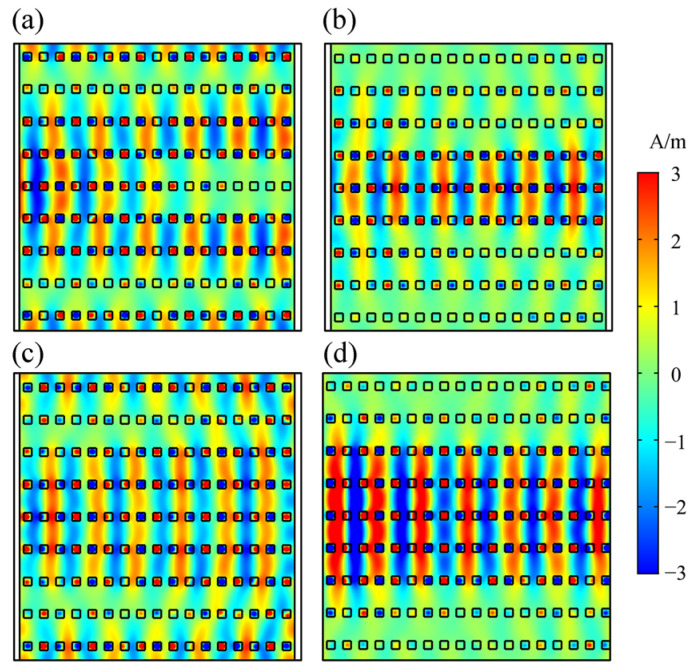
Light propagation in the HMM PhC membrane at different operating frequencies of (**a**) 550 THz, (**b**) 554.57 THz, (**c**) 560 THz, and (**d**) 554.57 THz. The observed beam behaviors at 550, 554.57, and 560 THz represent divergence, collimation, and convergence, respectively.

Figure 4 depicts light propagation in the HMM PhC membrane observed at different frequencies. Incident light was introduced on the left side of the PhC with a Gaussian shape, as shown in Figure 4, and the width of the beam was set to be 4*a* (Figure 4a,b) or 8*a* (Figure 4c,d). The wave propagation clearly indicates different beam behaviors of divergence (Figure 4a), collimation (Figure 4b,d), and convergence (Figure 4c). We used finite-size PhCs in the calculations, and the boundary condition is set as perfect match layer (PML); thus, the waves have no reflection on the boundary of the PhCs and air. Normally, divergence is accompanied by an increase in beam width and vice versa. However, because waves are reflected back and forth by rods inside the PhC, both beam broadening and narrowing result in the spreading of the field, as shown in Figure 4a,c. Thus, in the divergence and convergence situations (Figure 4a,c), neither broadening nor narrowing causes any transverse localization of the beam, and we can observe that the fields are continuously distributed throughout. In the collimation situation (Figure 4b,d), the modes are localized transversely, and the PhC now acts as a straight waveguide. Note that in Figure 4d, field intensity along the propagating path is decreasing. This is caused by the absorption of waves by the Ag layers. The material loss of Ag is no longer negligible at the operating frequencies. In the collimation situation, the beam shows transverse localization; therefore, the SC modes are also called spatial solitons [41]. As previously mentioned, the beam behavior around the SC point changes significantly with the parameters of the structure or the operating frequency. For instance, at the SC frequency, beams exhibit the SC effect, in which the beam width remains the same throughout the propagation. On the one hand, when the operating frequency is increased to a larger value, the beam behavior correspondingly changes to converge, as indicated by the equifrequency contours in Figure 3b. In contrast, when the operating frequency is decreased, the beam behavior is divergent. Thus, such an SC effect indicates immense potential for application in optical devices, such as nonlinear optical switches, filters, and tunable lenses.

**Figure 5:**
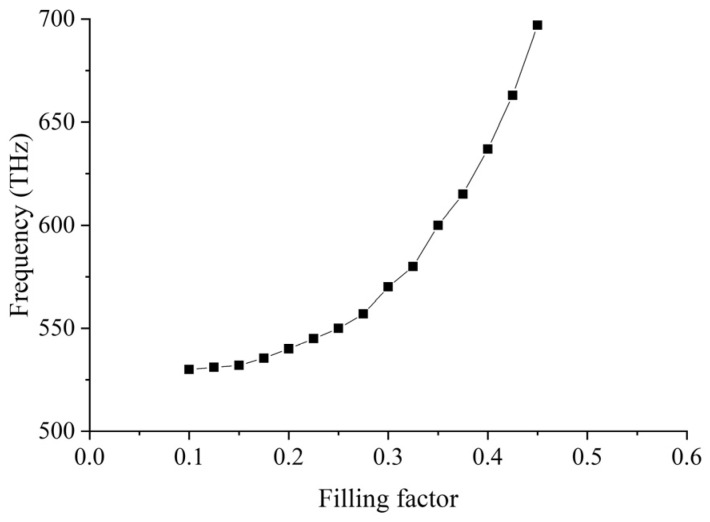
Correlation between changes in the position of SC points and HMM filling factor *f*.

Figure 5 shows the position of the SC point when the HMM has different filling factors. When the filling factor is 0.4, the SC frequency is 637 THz, with *ε_xx_* = −157.46 + 28.08*i*, *ε_yy_* = 6.27 + 0.10*i*, where the HMM is now functioning as Type I HMM. Both the structural parameters of the PhC membrane and HMM affect the position of the SC point. Thus, SC modes can be adjusted according to the requirements of applications in various situations. The SC point exists only when the filling factor is between 0.1 and 0.45.

### 3.2. Tunability of the HMM PhCs

HMMs exhibit immense potential for controlling the beam behavior in PhC systems. From Figure 2, it is understood that the effective permittivity of the HMM changes significantly according to different filling factors. Here, we investigate the effect of nonlinearity on the beam behavior in HMM PhC membranes. The strong field confinement of the HMM enhances the nonlinear effects; thus, the effective permittivity of the HMM changes according to the incident field intensity; accordingly, the beam behavior in PhCs can be modified.

**Figure 6:**
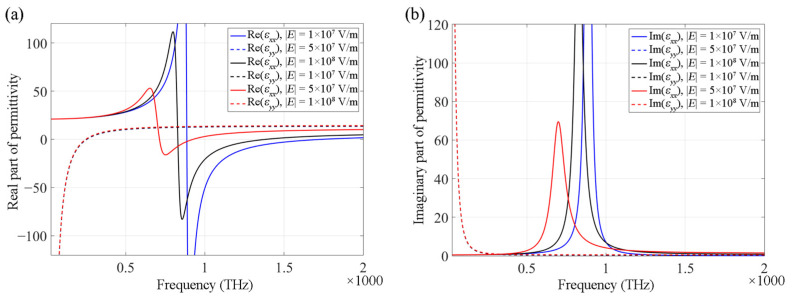
Change of transverse and longitudinal components of permittivity according to different field intensities in nonlinear regime. (**a**,**b**) are the real and imaginary parts of the permittivity, respectively.

The nonlinearities of the Ag and Si layers are different. The real part of the nonlinear susceptibility of Si is about 10^−10^ esu, while that of Ag is about 10^−8^ esu. With a certain incident power, the nonlinear effect of Si layers is negligible compared with the nonlinear effect of Ag layers. Because the nonlinearity of Ag is significantly larger than that of Si, we only consider the nonlinearity arising from the metal layers. The Kerr effect modifies the permittivity of the metal layers of HMMs in accordance with the relation

(6) $$\varepsilon_{\mathrm{NL}}=\varepsilon_{m}+\chi^{\left(3\right)}{\left|E\right|}^{2}$$

where *χ*^(3)^ is the third-order nonlinear optical susceptibility of Ag, and *E* is the electric field intensity. The nonlinear susceptibility of Ag is *χ*^(3)^ = 2.49 × 10^−8^ + 7.16 × 10^−9^*i* esu [42]. According to Equations (2) and (3), the transverse and longitudinal components of the effective permittivity of the HMM can be modified by changing the field intensity, as shown in Figure 6. As shown in Figure 6, the modification is highly efficient, particularly near the transferring point of the HMM. When changing the intensity of the incident light, owing to the permittivity change, the HMM could transfer between two types, for instance, from a Type I HMM to an effective dielectric. In the PhC system, this leads to a significant change in the dispersion properties of Bloch modes. In fact, a small change in the permittivity results in a notable change in the band structure. One reason is that the modes of the second band of the HMM PhC have fields concentrating inside the HMM rods, where they are also enhanced by the metamaterial; another reason is that the SC point of the band has a low-group-velocity. Note that in Figure 6, the dashed blue and dashed black lines have been covered by the dashed red line. This is because the change of *ε_xx_* is very small when field value changes. It also means that in HMMs, *ε_xx_* provides the main contribution to nonlinearity, and *ε_yy_* can be neglected.

Not only do HMMs generate unique modes, but HMMs can also amplify the nonlinear effect. Here, we compare the permittivity changes of bulk materials and HMMs at different incident powers. When the operating frequency is 554.57 THz and the filling factor is 0.2, by comparing the permittivity changes $\left(\varepsilon_{\mathrm{NL}}-\varepsilon \right)$ of Ag and HMM, we obtain the following: (a) when |**E**| = 1 ×10^7^ V/m, ${\left(\varepsilon_{\mathrm{NL}}-\varepsilon \right)}_{\mathrm{Ag}}$ = 0.035 + 0.010*i*, ${\left(\varepsilon_{\mathrm{NL}}-\varepsilon \right)}_{\mathrm{HMM},\;\varepsilon_{xx}}$ = 0.038 + 0.015*i*, ${\left(\varepsilon_{\mathrm{NL}}-\varepsilon \right)}_{\mathrm{HMM},\;\varepsilon_{yy}}$ = 0.007 + 0.002*i*; (b) when |**E**| = 5 × 107 V/m, ${\left(\varepsilon_{\mathrm{NL}}-\varepsilon \right)}_{\mathrm{Ag}}$ = 0.869 + 0.250*i*, ${\left(\varepsilon_{\mathrm{NL}}-\varepsilon \right)}_{\mathrm{HMM},\;\varepsilon_{xx}}$ = 1.035 + 0.439*i*, ${\left(\varepsilon_{\mathrm{NL}}-\varepsilon \right)}_{\mathrm{HMM},\;\varepsilon_{yy}}$ = 0.173 + 0.050*i*; and (c) when |**E**| = 1 × 10^8^ V/m, ${\left(\varepsilon_{\mathrm{NL}}-\varepsilon \right)}_{\mathrm{Ag}}$ = 3.477 + 0.010*i*, ${\left(\varepsilon_{\mathrm{NL}}-\varepsilon \right)}_{\mathrm{HMM},\;\varepsilon_{xx}}$ = 5.447 + 3.519*i*, ${\left(\varepsilon_{\mathrm{NL}}-\varepsilon \right)}_{\mathrm{HMM},\;\varepsilon_{yy}}$ = 0.695 + 0.200*i*. Thus, the nonlinear effect of the HMM is more efficient than the nonlinear effect of the bulk material of Ag. Compared to conventional bulk materials (such as Ag), HMM applied in PhC membranes could afford a larger tunability.

**Figure 7:**
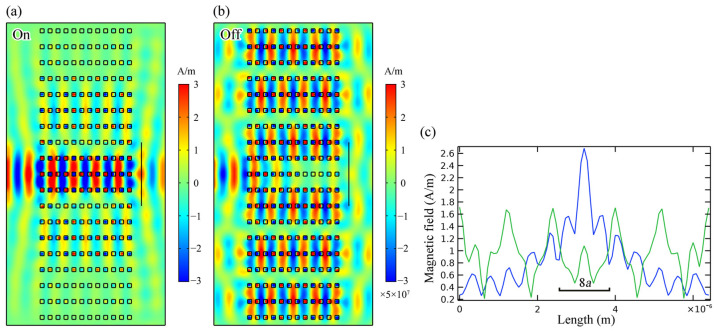
Nonlinear response of wave propagating in HMM PhC membranes as beam behavior changes from (**a**) collimation when |**E**| = 1 V/m (linear) to (**b**) divergence when |**E**| = 5 × 10^7^ V/m (nonlinear). (**c**) Magnetic field profiles along the solid line in (**a**,**b**), showing in blue and green, respectively.

Consider an incident light with frequency *f*_SC_ = 554.57 THz propagating in the PhCs with *f* = 0.2. By increasing the incident power, the beam behavior changes from collimation to divergence, as shown in Figure 7. In the figure, the incident light source is located on the left side of the PhC, and the source is Gaussian-shaped with a beam width of 4*a*. Light propagation in the PhCs shows collimation and divergence when |**E**| = 1 V/m and |E| = 5 × 10^7^ V/m, respectively. Such phenomena can be utilized as an all-optical switch or tunable lens. Considering the collimation modes of the HMM PhC membrane as “on” state and the non-collimation modes as “off” state, we obtained a nonlinear optical switch whose state could be modulated by the incident power. We measured the transmitted power on the right side of the PhCs, as shown by the solid line in Figure 7. The transmissions of the switch in the “on” and “off” states equal 68% and 17%, respectively. Note that if we use another frequency of 637 THz with *f* = 0.4, a larger field-intensity-sensitivity can be obtained because the effective permittivity of the HMM changes more significantly near the SC points. In this situation, owing to the transition of the HMM from an effective dielectric to Type I HMM, bandgap engineering of the PhCs would be more complicated, but SC effects in the 2D HMM PhCs would still be feasible, and one can expect more fascinating phenomena. Owing to the absorption of light by Ag, the transmission of the HMM PhC membrane switch in the “on” state is not very high (68%). This problem could be solved using materials that have less absorption of light, such as doped semiconductors.

## 4. Conclusions

In this study, we propose PhC membranes that contain HMM square rods and investigated their unique properties in the enhancement of the SC effect in PhCs. Such 2D systems are very suitable to be applied in controlling beam behaviors, which is a capability that 1D systems do not have. The dispersion properties and band structures of the 2D HMM PhCs were calculated, indicating that the 2D HMM PhC system can support an all-angle SC mode in the second band. Near the SC point, the dispersion curves change significantly, indicating that they are highly effective in changing the divergence, collimation, or convergence beam behaviors, and controlling light propagation. These are properties that a 1D system cannot achieve, which confirms the superiority of 2D structures. Moreover, the HMM PhC membranes demonstrate not only unique dispersion properties but also enhanced tunability owing to HMMs and SC effect of PhCs. Accordingly, compared to PhC membranes with conventional dielectric rods, HMM PhCs are more efficient in modulating the dispersion property and controlling the wave propagation. The tunability of HMM PhC membranes is large and suitable to be applied in an optical switch. Overall, HMM PhC membranes demonstrate great potential for use in compact integrated optical devices. In a large-scale system, the absorption of light by the Ag layers is expected to significantly affect the performance of the proposed optical switch. Accordingly, in future endeavors, we intend to focus on increasing the efficiency of HMM PhC membranes by reducing the material absorption. The prospect for future work will also focus on the enhancement of Si nonlinearity with the hyperbolic structure when the filling factor of Ag layers is very small, and the HMM can be treated as approximate Si.

## Data Availability

The data presented in this study are available on request from the corresponding author.
